# 5-Hydroxytryptamine Receptor Subtypes and their Modulators with Therapeutic Potentials

**DOI:** 10.4021/jocmr2009.05.1237

**Published:** 2009-06-21

**Authors:** Anand B. Pithadia, Sunita M. Jain

**Affiliations:** a Department of pharmacology, L.M. College of Pharmacy, Navrangpura, Ahmedabad-3800 09, India

## Abstract

5-hydroxytryptamine (5-HT) has become one of the most investigated and complex biogenic amines. The main receptors and their subtypes, e.g., 5-HTI (5-HT1A, 5-HT1B, 5-HTID, 5-HTIE and 5-HT1F), 5-HT2 (5-HT2A, 5-HT2B and 5-HT2C), 5-HT3, 5-HT4, 5-HT5 (5-HT5A, 5-HT5B), 5-HT6 and 5-HT7 have been identified. Specific drugs which are capable of either selectively stimulating or inhibiting these receptor subtypes are being designed. This has generated therapeutic potentials of 5-HT receptor modulators in a variety of disease conditions. Conditions where 5-HT receptor modulators have established their use with distinct efficacy and advantages include migraine, anxiety, psychosis, obesity and cancer therapy-induced vomiting by cytotoxic drugs and radiation. Discovery of 5-HT, its biosynthesis, metabolism, physiological role and the potential of 5-HT receptor modulators in various nervous, cardiovascular and gastrointestinal tract disorders, bone growth and micturition have been discussed in this article.

**Keywords:**

5-hydroxytryptamine (5-HT) receptors; Modulators; Biogenic amines

## Introduction

In 1930s, Erspamer began to study the distribution of enterochromaffin cells, which stained with a reagent for indoles. The highest concentrations were found in gastrointestinal mucosa, followed by the platelets and the CNS. Hence the unknown indole was named entramine. Page and his colleges at Cleveland clinic isolate and characterize a vasoconstrictor substance released from clotting blood. The substance was called serotonin (1948). Rapport deducted that the active moiety was 5-hydroxy tryptamine, which was isolated as serotonin by Page. In 1952 Erspamer and Asero identified entramine as 5-HT. 5-HT is autacoids as well as important neurotransmitter in CNS and PNS The neuron that secrets 5-HT are termed as seretonergic neurons. The seretonergic system is known to modulate mood, emotion, sleep and so it is implicated in the control of numerous behavioral and physiological functions. All functions are involving various receptors. Fruits like banana, tomatoes, nuts, plumps, venoms of bees and wasps also contain 5-HT [1].

## Biosynthesis and Metabolism Pathway and Distribution

5-HT or Serotonin is biosynthesized from tryptophan amino acid. Tryptophan is converted to 5 hydroxy tryptophan by tryptophan hydroxylase which by action of dopa decarboxylase converted into serotonin. This synthesized serotonin is mainly stored in chramaffin and enteric neurons (90%). This biosynthesis not occur in CNS and Platelet but they take up 5-HT from circulation. 5-HT is metabolized by monoamine oxidase enzyme (MAO) to 5 Hydroxyindole acetaldehyde, which through aldehyde dehydrogenase converted into 5-Hydroxyindole Acetic Acid (5-HIAA). This 5-HIAA serve as marker for Malignant carcinoid syndrome in which higher concentration of 5-HT in body lead to 20 fold higher excretion of 5-HIAA. 5-HT is converted into N-acetyl 5-HT through enzyme 5-HT N-acetylase which is with help of hydroxyl indole c-methyl transferase converted into melatonin. Melatonin is an important hormone that maintains sleep cycle and also acts as antioxidant [2].

### Physiological Role of Serotonin

Serotonin (5-hydroxytryptamine) is principally found stored in three main cell types - (a) serotonergic neurons in the CNS and in the intestinal myenteric plexus, (b) enterochromaffin cells in the mucosa of the gastrointestinal tract and (c) in blood platelets. Serotonergic neurons and enterochromaffin cells can synthesize serotonin from its precursor amino acid L-tryptophan, whereas platelets rely upon uptake of serotonin for their stores. Likewise, serotonergic neurons also have the capacity for amine uptake via serotonin transporters. In central nervous system serotonin acts as neurotransmitter as well as precursor for melatonin hormone synthesis in pineal gland. It regulates gastrointestinal motility and involved in haemostasis on platelets. The net effect of 5-HT is to cause platelet aggregation. It causes bronchoconstiction, positive inotropic and action on heart. All these actions are brought about by it's interaction with various membrane receptors.

### 5-HT Receptor Subtypes

Gaddum and Picarelli in 1957 first suggested that 5-HT receptors located on guinea pig ileum smooth muscle cells could be blocked by dibenzyline and the serotonin mediated depolarization of intramural cholinergic neuron could be blocked by Morphine. They therefore classified 5-HT receptors as "D" and "M" subtypes Subsequent research demonstrated that certain action of 5-HT like vasoconstriction in the carotid vessels could neither be blocked by dibenzyline nor by morphine such reports initiated search for other "Non D, Non M" receptors [3]. Peroutka and Snyder (1979) used radioligand binding study to classify 5-HT receptor subtypes. However the classification scheme proved to be invalid. Hence widely accepted classification scheme is based on pharmacological properties, second messenger function and deducted amino acid sequence [4]. This classification scheme proposes 7 subfamilies of 5-HT receptors.

Table 1a, b, and c give information about the seretonergic receptor subtypes with their signal transduction mechanism, their location, physiological action, agonist and antagonists.

**Table 1a:** Seretonergic receptor subtypes

Receptor Subtype	Second Messenger	Location	Physiological Action	Agonist	Antagonist
5-HT_1A_	Inhibit adenylate cyclase and activate receptor operated K^+^ channel. Inhibit voltage gated Ca^2+^ channel	CNS: Raphe nuclei, Hippocampus PNS: Cholinergic heteroreceptor in myenteric plexus	1. Seretonergic auto receptor 2. Neuronal inhibition 3. Facilitate Ach and nor adrenaline release 4. Cholinergic nerve terminal in myenteric plexus 5. Hyperphagia (led to obesity)	8-OH-DPAT Buspirone (PA) Ipsapirone Flesinoxan 5-CT Quetiapine	WAY100635 5-F 8-OH DPAT Spiperone Sibutramine
5-HT_1B_	Inhibit adenylate cyclase	CNS: subiculum substania nigra PNS: Vascular smooth muscle	1. Seretonergic auto receptor 2. Terminal heteroreceptor to control release of Ach and nor adrenaline 3. Contraction of vascular smooth muscle	5-CT 8-OH-DPAT Sumatriptan Ergotamine (PA)	GR55562 SB224289 SB236057 Methiothepin Cynopindolol
5-HT_1D_	Inhibit adenylate cyclase	CNS: cranial blood vessel PNS: Vascular smooth muscle	1. Seretonergic auto receptor 2. GABAergic and Cholinergic heteroreceptor 3. Vasoconstriction of intracranial blood vessel smooth muscle	Sumatriptan Zolmitriptan Nortriptan L694247 Ergotamine (PA)	Methiothepin Ergotamine BRL15572
5-HT_1E_	Inhibit adenylate cyclase	CNS: cortex striatum PNS: m-RNA in vascular tissue	Unknown	5-CT (weak agonist) 5-HT	Methiothepin
5-HT_1F_	Inhibit adenylate cyclase	CNS: Spinal cord hippocampus PNS: Uterus, mesentery, vascular smooth muscle	Trigeminal (V) neuro inhibition in guinea pig and rat	No selective agonist or antagonist are available	

8-OH DPAT, 8-Hydroxy-2-(di-n-propylamino) Tetraline; PA, Partial Agonist; LSD, Lysergic Acid Diethylamide; 5-CT, 5 Carboxamidotryptamine; CSF, Cerebrospinal Fluid; 5-HT, 5 Hydroxytryptamine; CTZ, Chemoreceptor Trigger Zone

**Table 1b:** Seretonergic receptor subtypes

Receptor Subtype	Second Messenger	Location	Physiological Action	Agonist	Antagonist
5-HT_2A_ D receptor	Phospholipase C activation	CNS: cerebral cortex PNS: GI, vascular and bronchial smooth muscle, platelets	1. Neuro excitation 2. Broncho constriction 3. Platelet aggregation 4. Smooth muscle contraction	α-methyl 5-HT 5-CT Sumatriptan 8-OH DPAT LSD	Ketanserin Cyproheptadin Pizotifin Methylsergide Risperidone Olanzapine Clozapine
5-HT_2B_	Phospholipase C activation	CNS: cerebellum hypothalamus PNS: Vascular endothelium, stomach	Endothelium dependant vaso relaxation via NO production and stomach fundus contraction	5-CT Sumatriptan BW723C86	RS127445 SB204741
5-HT_2C_	Phospholipase C activation	CNS: choroid plexus, hippocampus, hypothala-mus	Modulation of transferin production and modulation of CSF volume	α-methyl 5-HT 5-CT Quipazine	Methylsergide Olanzapine Mesulergine
5-HT_3_ M receptor	Ligand gated ion channel	CNS: area postrema PNS: Abdominal visceral afferent neuron	1. Stimulate vomiting by acting on CTZ and by vagal neuro excitation 2. Stimulate nociceptive (pain mediating) nerve ending led to pain	2-me5-HT 5-MeOT	Ondensetron Tropisetron Granisetron
5-HT_4_	Activation of adenylate cyclase	CNS: Hippocampus PNS: GIT	Neuronal excitation Increase GI motility	Mosapride Cisapride Zacopride	GR113808 SB204070
5-HT_5A_	Unknown	CNS: olfactory bulb, Hebenula	Unknown	No selective agonist or antagonist are available	
5-HT_5B_	Unknown	CNS: olfactory bulb, Hebenula	Unknown	No selective agonist or antagonist are available	

8-OH DPAT, 8-Hydroxy-2-(di-n-propylamino) Tetraline; PA, Partial Agonist; LSD, Lysergic Acid Diethylamide; 5-CT, 5 Carboxamidotryptamine; CSF, Cerebrospinal Fluid; 5-HT, 5 Hydroxytryptamine; CTZ, Chemoreceptor Trigger Zone

**Table 1c:** Seretonergic receptor subtypes

Receptor Subtype	Second Messenger	Location	Physiological Action	Agonist	Antagonist
5-HT_6_	Activation of adenylate cyclase	CNS: caudate putamen, hippocampus PNS: Superior cervical ganglia	Modulation of CNS Ach release	No selective agonist available	SB271046 Methiothepin
5-HT_7_	Activation of adenylate cyclase	CNS: hypothalamus PNS: gastrointestinal and vascular smooth muscle	Smooth muscle relaxation	5-HT Sumatriptan 8-OH DPAT	SB258719 Methiothepin

8-OH DPAT, 8-Hydroxy-2-(di-n-propylamino) Tetraline; PA, Partial Agonist; LSD, Lysergic Acid Diethylamide; 5-CT, 5 Carboxamidotryptamine; CSF, Cerebrospinal Fluid; 5-HT, 5 Hydroxytryptamine; CTZ, Chemoreceptor Trigger Zone

## Theapeutic Uses of Drugs Acting on Serotonin Receptors

The various indications where 5-HT receptor modulators have been reported to be of beneficial are given below.

### Central Nervous System

#### Depression

The hypothesis in affective disorders focuses on an involvement of neurotransmitters noradrenaline (norepinephrine), 5-HT and dopamine. It has been found that some depressed patients appear to have reduced cerebral concentration of 5-HIAA (5-Hydroxy indole acetic acid) (the metabolite of 5-HT), whereas others appear to have reduced level of methoxyhydroxyphenylglycerol (MHPG), a metabolite of noradrenaline. More consistent changes have been reported in the plasma concentration of L-tryptophan (the precursor for serotonin). The classical mechanism of antidepressant drugs is by increasing effective synaptic concentration of monoamines-NE, 5-HT and dopamine, either by blocking the oxidative enzyme in synaptic terminals that degrade these monoamines (e.g. MAO Inhibitors) or by blocking the reuptake of these transmitters i.e., reuptake blockers. It has been shown that many antidepressant drugs like imipramine,desipramine, amitriptyline, nortriptyline, doxepin, amoxapine, maprotiline, mianserin and trazodone are also antagonists at 5-HT1c receptors in the brain. Selective 5-HT reuptake inhibitors, e.g., fluoxetine, fluoxamine, paroxetine, citalopram and sertraline are effective as tricyclic anti-depressants (TCAs) and MAO-I (monoamine oxidase inhibitors) in treating depression of moderate degree but probably less effective than TCAs in treating severe depression [5].

#### Psychosis

The idea that 5-HT dysfunction could be involved in schizophrenia was based on the fact that LSD (Lysergic acid diethylamide) produce schizophrenia like symptoms. Many effective anti-psychotic drugs in addition to blocking dopamine receptors, also act as 5-HT receptors antagonists. Clozapine, an atypical anti-psychotic drug has more effect on limbic system and 5-HT2 receptors, which may explain its reduce risk of extrapyramidal symptoms [6]. Risperidone, which blocks both 5-HT2 and D2 receptors does improve both positive and negative symptoms of schizophrenia, while ritanserin, a very potent and selective 5-HT2 receptor antagonist, showed significant improvement in Type II schizophrenics [those with primarily negative symptoms].Drugs acting on 5-HT3 receptors e.g. ondansetron have also been investigated as new anti-psychotics [7]. Further studies are required to prove their usefulness or otherwise.

#### Migraine

5-HT1B and 5-HT1D receptors are found mainly as presynaptic inhibitory receptors in basal ganglia. 5-HT1D receptor subtype which is expressed in cerebral blood vessels is believed to be involved in migraine. Sumatriptan, 5-HT1D receptor agonist is used to treat acute attack of migraine. It constrict large arteries and inhibit trigeminal nerve transmission. Sumatriptan cause pain at site of injection and also cause hypertension, so contraindicated to patient with IHD (Ischemic Heart Disease) while zolmitriptan is fast acting and don't cause chest pain. Naratriptan,eletriptan, almotriptan and rizatriptan are other agonists of 5-HT1D and 5-HT1B receptors, active as antimigraine agents at lower dose than sumatriptan. They have properties similar to those of sumatripan but a better bioavailability by oral route and are presented in the form of tablets. Their therapeutic use is also the treatment of migraine attacks. 5-HT2 receptor antagonists e.g. dihydroergotamine, methysergide, pizotifen and cyproheptadine are mainly use for migraine prophylaxis [8]. Methysergide is rarely used because of development of "Retroperitoneal fibrosis" or "Ormond's disease" which is characterize by development of fibrotic mass in peritoneal cavity like kidney. Cyproheptadine in addition to 5-HT2A blocking activity also has anti-allergic action due to histamine receptor antagonistic activity antimuscarinic and Ca^+2^ antagonistic activities. It is used in children to enhance appetite and also reduce dumping after gastrin surgery (Post Gastractomy Dumping Syndrome) [9].

#### Pain

5-HT stimulates nociceptive (pain mediating) sensory nerve ending, an effect mediated by 5-HT3 receptors. Thus 5-HT3 receptors could play a role in nociception at spinal level [10]. It is further reported that 5-HT3 receptor stimulation in the spinal cord results in GABA release that may inhibit nociceptive signal transmission at sites post-synaptic to primary afferent terminals. These findings may herald the development of new non-opioid, non- addictive analgesics. The relief by 5-HT3 receptor antagonists in migraine and visceral discomfort associated with irritable bowel syndrome is known [11]. Further clinical evaluation is however needed to establish this concept. There is considerable evidence of a role for 5-HT mediation in cardiac pain. It has been suggested that combined antagonism of 5HT2 and 5-HT3 receptors may provide more effective therapy for the treatment of angina [12].

#### Anxiety

Buspirone is a partial agonist at 5-HT1A receptors used to treat various anxiety disorders.It shows high specific for 5-HT1A receptors, which are inhibitory autoreceptors that reduce the release of 5-HT and other mediators. Buspirone and related compounds ipsapirone and gepirone don't cause sedation or motor in coordination nor have withdrawal effects as with other anxiolytics like barbiturates [13].

#### Parkinsonism

Of the three cardinal symptoms of parkinsonism, i.e., rigidity, tremor and bradykinesia, tremor may be mediated by 5-HT2 receptors. This was revealed by the success of ritanserin, a potent and selective 5-HT2 receptor antagonist, in reducing the tremor of parkinsonism patients [14].

#### Treatment of drug abuse

5HT3 receptor antagonism has also been shown to reduce the alcohol intake in animals and in human .However, more preclinical and clinical studies are required, to arrive at any meaningful conclusion about the usefulness of 5-HT3 receptor blockers in treatment of drug abuse [15].

#### Tempreture regulation

Changes in temperature were determined following injection of noradrenaline, adrenaline, isoprenaline, dopamine and 5-hydroxytryptamine (5-HT) into the cerebral ventricles of the conscious mouse. 5-HT (10-160 μg) caused a fall in body temperature. The activity may be involving 5-HT2 receptor.Hence 5-HT could be the effective target to control body temperature [16].

### Anti-emetic Action

The central neural regulation of vomiting is vested in two separate units in medulla. These are vomiting centers and chemoreceptor trigger zone(CTZ).Impulses from CTZ pass to vomiting centre and integrate the visceral and somatic functions involved in vomiting .The main neurotransmitters considered to be involved in the control of vomiting are acetylcholine, dopamine, histamine and 5-HT.Receptors for these neurotransmitters have been demonstrated in relevant areas.5-HT3 receptors in brain particularly in the area postrema, a region of medulla in the vomiting reflex, and selective 5-HT3 receptor antagonists are useful as anti-emetic drugs [17]. Ondansetron, tropisetron and dolasetron are of particular value in preventing and treating vomiting cause either by radiation therapy in cancer patients or by administration of cytotoxic drugs such as cisplatin [17].

### Gastrointestinal tract

5-HTIA, 5-HTlc, 5-HT2, 5-HT3 and 5-HT4 receptors have been identified in the gut, in either the enteric nervous system or on smooth muscles [18-20]. The actions of 5-HTI-like receptors may include inhibition of release and smooth muscle contraction. 5-HT2 receptors located on the smooth muscle cells, when stimulated directly cause contraction of gastrointestinal smooth muscle and gut vascular smooth muscle. Some selective 5-HT2 receptor agonists stimulate contraction of the lower oesophageal sphincter [21]. 5-HT3 receptors are located on post-synaptic enteric and sensory neurones, on enteric neuronal membranes, in the vagus, on gastric endocrine glands and in the CNS. They are implicated in the modulation of cholinergic transmission in the enteric nervous system, where their stimulation has been reported to facilitate acetylcholine (ACh) release. 5-HT4 receptors are believed to be located in the nerve terminals on both cholinergic interneurones and motor neurones. Their stimulation leads to increased release of ACh and they accelerate upper gastrointestinal transit as well as increase in colonic motor activity [22].

#### Irritable bowel syndrome (IBS)

5-HT4 agonists increase intestinal motility and could be used in the treatment of gastroesophageal reflux, intestinal paresis (constipation), irritable bowel syndrome. The first drug of this group is tegaserod. A frequent adverse effect of tegaserod is diarrhea and a rare more severe effect is ischemic colitis. Depending on whether diarrhoea or constipation is the presenting problem, IBS is sub-classified as either diarrhoea predominant IBS or constipation predominant IBS. Though the exact pathophysiology remains unclear, it has been reported that patients of IBS have a higher resting tone of the intestinal smooth muscles and have an excessively sensitive colon. Patients with IBS may also in some cases have a reduced tolerance to gas infusion into the small bowels and the threshold for perception of intestinal contraction may be lower than normal [23]. 5-HT3 receptor blockade has been shown to slow colonic transit in healthy volunteers and has also been reported to reduce visceral hypersensitivity [24].Thus 5-HT3 receptor antagonists like ondansetron were thought to be of benefit in diarrhea predominant IBS cases. Stimulation of 5HT4 receptors facilitate cholinergic neurotransmission in the gut and thereby increase colonic motor activity. Thus, 5HT4 receptor agonists like cisapride, zacopride, renzapride have a potential role in the constipation predominant IBS patients [25].

#### Malignant carcinoid syndrome

Carcinoid syndrome is a rare disorder associated with malignant tumors enterochrommafin cells, usually arising in the small intestine and metastatising to liver. These tumors secreate variety of hormones. 5-HT is the important one. The syndrome is readily diagnosed by measuring excretion of 5-HIAA(5-hydroxyindole acetic acid);the main metabolite of 5-HT in the urine. The concentration of which increase up to 20-fold. 5-HT2 antagonists such as cyproheptadine are effective in controlling some of the symptoms of carcinoid syndrome. A complemantary therapeutic approach is to use a long acting analogue of somatostatin analogue, namely octreotide, which suppress the hormone secretion from various neuroendocrine cells, including carcinoid cells [26].

#### Dyspepsia

Dyspepsia is defined as pain or discomfort centered in the upper abdomen in the absence of any structural or biochemical abnormality. 5-HT3 receptor antagonists have been reported to reduce visceral pain reflex in the gut, and studies in the rat showed that granisetron and tropisetron (but not ondansetron) reduced the pain response induced by duodenal distension [27]. Thus 5-HT3 receptor antagonists would theoretically benefit patients of dyspepsia who have increased visceral sensitivity.

#### Non-cardiac chest pain

Sometimes referred to as chest pain of undetermined etiology (CPUE), it is an ill defined entity requiring urgent elimination of other differential diagnosis. Some authors have reported that visceral nociceptive abnormalities in the oesophagus may contribute in the etiopathogenesiss of CPUE [28]. As 5-HT3 receptor antagonists can reduce the visceral pain reflex in the gut, they would theoretically be of benefit in the management of such cases.

#### Gastro-oesophageal reflux disease

The symptoms of pain and anxiety, seen in gastrooesophageal reflux disease (GERD) are due to a pathological acid reflux into the oesophagus which may result from a combination of decreased lower oesophageal sphincter tone and impaired acid clearance [29, 30]. 5-HT4 receptor agonists having prokinetic action have been found clinically useful in such conditions.

### Cardiovascular system

Ketanserin, a 5-HT2 receptor antagonist with high affinity for peripheral 5-HT2 sites, reduces blood pressure by causing vasodilation and reducing total peripheral resistance. The reflex tachycardia seen with other vasodilators is not seen with ketanserin [31].This 5-HT2 receptor blockade may be very useful in protecting the microcirculatory bed against the detrimental effects of serotonin, which is massively released by aggregation of platelets, particularly when the vascular bed is predamaged by atherosclerosis, diabetes mellitus and old age. Ketanserin has also been reported to be more effective in the elderly [32].

### Ophthalmology

5-HT receptor modulators may have some potential in the treatment of ocular conditions such as glaucoma. A single topical application of 0.5 % ketanserin, a 5-HT2 receptor antagonist with additional alpha-l adrenoceptor blocking activity, has recently been reported to lower intra-ocular pressure (IOP) for 6-8 hours. This decrease in IOP was due to increased outflow and was not accompanied by any change in systolic or diastolic blood pressure, heart rate, pupil size, corneal thickness or tear secretion [33].

### Diabetes

**Figure 1:**
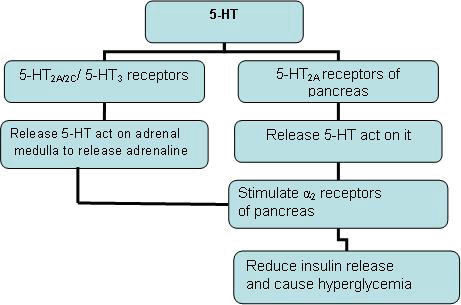
5-HT causes hyperglycemia.

In overnight fasted rats 5-HT was found to produce dose dependant increase in serum glucose level.5-HT may cause hyperglycemia as shown in Figure 1. It has been reported that 5-HT2A receptor agonist α methyl 5-HT increase serum glucose while 5-HT2A receptor antagonist sarprogralate reduce serum glucose level. In same study 5-HT3 receptor agonist 1-Phenyl biguanide potentiate hyperglycemia effect of 5-HT while 5-HT receptor antagonist ondensetron inhibit the same action [34, 35].

### Obesity

Sibutramine is an inhibitor of 5-HT/Noradrenaline reuptake at the hypothalamic sites that regulate food intake. Sibutramine reduce food intake and cause dose dependant weight loss, the weight loss being associated with decrease in obesity related risk factors [36, 37].

### Bone growth

Selective serotonin-reuptake inhibitors (SSRIs) antagonize the serotonin (5-hydroxytryptamine) transporter (5-HTT), and are frequently prescribed to children and adolescents to treat depression. However, recent findings of functional serotonergic pathways in bone cells and preliminary clinical evidence demonstrating detrimental effects of SSRIs on bone growth. The current work investigated the impact of 5-HTT inhibition on the skeleton in: (a) mice with a null mutation in the gene encoding for the 5-HTT; and (b) growing mice treated with a SSRI. In both models, 5-HTT inhibition had significant detrimental effects on bone mineral accrual. 5-HTT null mutant mice had a consistent skeletal phenotype of reduced mass, altered architecture, and inferior mechanical properties, whereas bone mineral accrual was impaired in growing mice treated with a SSRI. These phenotypes resulted from a reduction in bone formation without an increase in bone resorption and were not influenced by effects on skeletal mechanosensitivity or serum biochemistries. These findings indicate a role for the 5-HTT in the regulation of bone accrual in the growing skeleton and point to a need for further research into the prescription of SSRIs to children and adolescents [38].

### Micturition

Traditionally, central 5-HT-pathways are considered to be inhibitory in the control of micturition. However, at least in the rat, 5-HT1A and 5-HT7 receptors have excitatory actions. The use of antagonists for these two receptors indicates that both play an essential role in micturition in the rat and probably in the guinea pig. Interestingly, both receptors seem to have a similar role supraspinally, although they have opposing effects on adenylyl cyclase. The paucity of evidence compared with rat indicating that 5-HT plays an important role in the control of micturition may just reflect the lack of experiments carried out in this species. Overall the data indicate that 5-HT is an important transmitter involved in the control of micturition. However, further experiments are required to elucidate its precise role and the seeming difference in importance it has in this function between species [39].

## Conclusions

Basic scientific research has expanded at a great pace, and more and more potent and selective agonists and antagonists for different 5-HT receptor subtypes are discovered. The study at molecular level revealing more subtypes of each receptor family. Scientists are finding precise and specific involvement of such receptor subtypes in different physiological processes and pathological states. Therefore, drugs acting specifically on 5-HT, suggesting their therapeutic potentials in conditions either in CNS or in peripheral tissues. The 5-HT receptor modulating drugs have now established their therapeutic role in various disease conditions like emesis, anxiety and migraine, in various other neurological conditions , as well as peripheral disorders. More studies in future will guide therapeutic potential of 5-HT modulating drugs in other conditions.
